# LukMF′ is the major secreted leukocidin of bovine *Staphylococcus aureus* and is produced *in vivo* during bovine mastitis

**DOI:** 10.1038/srep37759

**Published:** 2016-11-25

**Authors:** Manouk Vrieling, Eveline M. Boerhout, Glenn F. van Wigcheren, Kirsten J. Koymans, Tanja G. Mols-Vorstermans, Carla J. C. de Haas, Piet C. Aerts, Ineke J. J. M. Daemen, Kok P. M. van Kessel, Ad P. Koets, Victor P. M. G. Rutten, Piet J.M. Nuijten, Jos A. G. van Strijp, Lindert Benedictus

**Affiliations:** 1 Department of Medical Microbiology, University Medical Center Utrecht, PO G04.614, Heidelberglaan 100, 3584 CX, Utrecht, The Netherlands; 2 Department of Infectious Diseases and Immunology, Faculty of Veterinary Medicine, Utrecht University, Yalelaan 1, 3584 CL Utrecht, The Netherlands; 3 Ruminant Research and Development, MSD Animal Health, Wim de Körverstraat 35, 5830 AA Boxmeer, The Netherlands; 4 Department of Farm Animal Health, Faculty of Veterinary Medicine, Utrecht University, Yalelaan 7, 3584 CL Utrecht, The Netherlands; 5 Department of Bacteriology and Epidemiology, Central Veterinary Institute part of Wageningen UR, Edelhertweg 15, 8219 PH Lelystad, The Netherlands; 6 Department of Veterinary Tropical Diseases, Faculty of Veterinary Science, University of Pretoria, Private Bag X04, Onderstepoort 0110, South Africa

## Abstract

*Staphylococcus aureus* is a major human and animal pathogen and a common cause of mastitis in cattle. *S. aureus* secretes several leukocidins that target bovine neutrophils, crucial effector cells in the defence against bacterial pathogens. In this study, we investigated the role of staphylococcal leukocidins in the pathogenesis of bovine *S. aureus* disease. We show that LukAB, in contrast to the γ-hemolysins, LukED, and LukMF′, was unable to kill bovine neutrophils, and identified CXCR2 as a bovine receptor for HlgAB and LukED. Furthermore, we assessed functional leukocidin secretion by bovine mastitis isolates and observed that, although leukocidin production was strain dependent, LukMF′ was most abundantly secreted and the major toxin killing bovine neutrophils. To determine the role of LukMF′ in bovine mastitis, cattle were challenged with high (S1444) or intermediate (S1449, S1463) LukMF′-producing isolates. Only animals infected with S1444 developed severe clinical symptoms. Importantly, LukM was produced *in vivo* during the course of infection and levels in milk were associated with the severity of mastitis. Altogether, these findings underline the importance of LukMF′ as a virulence factor and support the development of therapeutic approaches targeting LukMF′ to control *S. aureus* mastitis in cattle.

*Staphylococcus aureus* is a common opportunistic pathogen that can cause a broad array of diseases in humans and animals^1^,^2^. In dairy cattle, *S. aureus* is a major cause of mastitis and responsible for reduced animal welfare and large economic losses. Its pathogenicity is linked to its ability to secrete a vast number of virulence factors, among which secreted toxins play an eminent role^3^. The bicomponent pore forming toxins, also named leukocidins or leukotoxins, are highly effective killers of phagocytes^4^. They are secreted as two monomers, the S- and F-components, of which the S-subunit binds to a specific proteinaceous receptor on the cell surface. Subsequent recruitment of the F-component and oligomerization of alternating S- and F- components results in formation of octameric pores in the cell membrane eventually leading to cell death^5^. *S. aureus* secretes several other factors able to kill leukocytes, (e.g. the pore forming alpha-toxin, spingomyelinase, phenol-soluble modulins)^6^, which may act synergistically with bicomponent leukocidins^7^,^8^, adding to the leukotoxic action of *S. aureus*. Phagocytes are important effector cells in the first line of defence against *S. aureus*^9^ and the selective killing of phagocytes by *S. aureus* leukocidins has been shown to be detrimental to survival of the host^10^,^11^. In bovine mastitis, prompt neutrophil recruitment is key to limit *S. aureus* infections^12^. Although several leukocidins have been shown to successfully target and kill bovine neutrophils^13^, their expression by bovine mastitis isolates and their role in the pathogenesis of bovine *S. aureus* mastitis is unknown.

*S. aureus* isolates can harbour up to six leukocidins, among which the γ-hemolysins (HlgAB and HlgCB) and LukAB (also known as LukGH) are most common, because of their location in the core genome^4^. LukED is encoded on the common pathogenicity island νSaβ and is present in most strains^14^. However, strains from the ruminant lineage CC133 encode a premature stop codon in LukE, which prevents the formation of functional LukED^15^. In addition, *S. aureus* can acquire two phage encoded leukocidins, Panton Valentine Leukocidin (PVL) and LukMF′. While the PVL genes are restricted to human strains, LukMF′ is associated with animal strains, especially with isolates from bovine mastitis^16^,^17^. Reported prevalence of the *lukMF*′ operon in bovine isolates ranges from 10–86% and differs per geographic region^16^,^17^,^18^,^19^,^20^. LukMF′ has a very profound lytic effect on bovine neutrophils and monocytes^21^. For the bovine mastitis isolate S1444, it was shown that *S. aureus* employs LukMF′ to kill bovine neutrophils at a distance, thereby preventing phagocytosis^21^. Altogether, LukMF′ is hypothesized to be an important virulence factor in bovine mastitis.

In recent years, the species and cell specificity of the leukocidins have been clarified by identification of their host receptors. While LukAB interacts with the integrin CD11b^22^, all other leukocidins bind chemokine receptors. HlgAB and LukED both target CXCR1 and CXCR2. Additionally, HlgAB also interacts with CCR2 and LukED with CCR5^10^,^11^,^23^. HlgCB and PVL target C5aR1 and C5aR2, whereas LukMF′ specifically kills CCR1, CCR2, and CCR5 expressing cells^21^,^24^. Interspecies differences in receptor sequence, structure, and expression levels account for the observed species specificity of the host-toxin interactions^21^,^25^. While LukMF′ and HlgCB have been shown to target both human and bovine orthologues of the same receptors^21^,^25^, the bovine targets of HlgAB, LukED, and LukAB have not been described.

Bovine mastitis isolates of *S. aureus* can potentially secrete five different leukocidin pairs, of which four have been described to target bovine neutrophils, i.e. LukMF′, LukED, HlgAB, and HlgCB^13^,^21^,^25^. The ability of LukAB, the fifth leukocidin, to kill bovine neutrophils has not been investigated. In this study we set out to characterise the role of different leukocidins in the pathogenesis of bovine *S. aureus* mastitis. First we assessed the toxic activity of LukAB on bovine neutrophils and identified the bovine target receptor orthologues of HlgAB and LukED. In order to elucidate whether bovine *S. aureus* strains secrete functional levels of each leukocidin, we measured their *in vitro* secretion by 10 bovine mastitis isolates. Next, we investigated the killing of neutrophils by secreted leukocidins *in vitro*. Finally, we studied the role of LukMF′ *in vivo* in an experimental intramammary *S. aureus* infection model.

## Results

### Identification of bovine target receptor orthologues of *S. aureus* leukocidins

**Figure 1:**
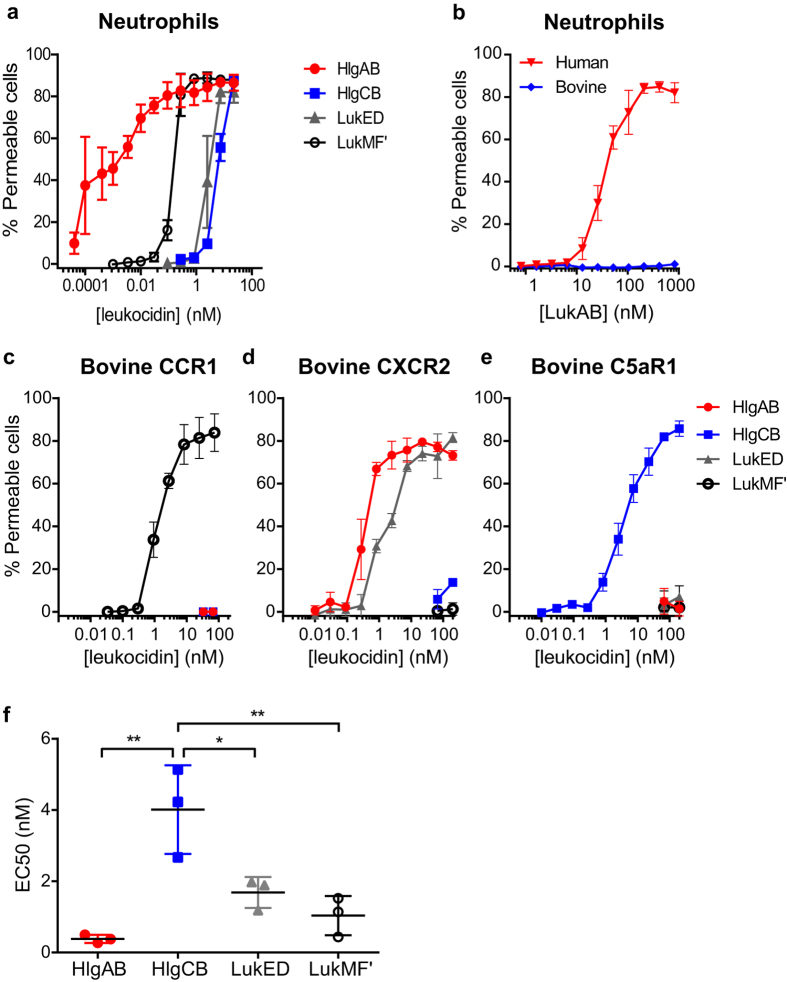
Specificity of leukocidins of *S. aureus* towards the bovine immune system. Bovine neutrophils were incubated with HlgAB, HlgCB, LukED and LukMF′ and pore formation was measured (**a**). Human and Bovine neutrophils were incubated with LukAB and pore formation was measured (**b**). HEK293T cells stably transfected with plasmids encoding bovine CCR1 (**c**), CXCR2 (**d**), and C5aR1 (**e**) were analysed for pore formation upon incubation with HlgAB, HlgCB, LukMF′, and LukED. Mean percentages of permeable cells ± standard deviation (SD) are shown (n = 3). (**f**) Half-maximal lytic concentrations (50% effective concentration [EC50]) of each bicomponent leukocodin were calculated for HEK293T cells expressing their cognate receptor (CXCR2, C5aR1, CXCR2, and CCR1 for HlgAB, HlgCB, LukED and LukMF′, respectively) and compared using a One-Way ANOVA followed by Tukey’s post-hoc test. *P ≤ 0.05, **P ≤ 0.01.

First we assessed the ability of the different leukocidins to permeabilise bovine neutrophils. Pore formation was induced by LukMF′, LukED and both γ-hemolysins (HlgAB and HlgCB) (Fig. 1a), but whereas LukAB permeabilised human neutrophils, bovine neutrophils were unaffected by the toxin (Fig. 1b) and, therefore, putative bovine receptor orthologues of LukAB were not further investigated. HEK293T cells were transiently transfected with an array of bovine chemokine receptors and screened for pore formation upon incubation with the γ-hemolysins (Supplementary Table S1). This screening revealed that bovine CXCR2 is a major target receptor for HlgAB, in addition to bovine CXCR4, and CCR2. Bovine CXCR2 and CCR5 were identified as targets for LukED. Next, bovine CCR1, CXCR2, and C5aR1 were stably expressed on HEK293T cells, cells were exposed to the different leukocidins and the half-maximal lytic concentrations (50% effective concentration [EC50]) were calculated (Fig. 1c,d,e). These values were subsequently compared (Fig. 1f) and while the EC50s for HlgAB and LukED treated CXCR2 and LukMF′ treated CCR1 expressing cells were comparable, the EC50 for HlgCB treated C5aR1 expressing cells was 3.9 fold higher than the average EC50 of the other leukocidins.

### LukMF′ is the most abundantly secreted leukocidin of *S. aureus* mastitis isolates

**Figure 2:**
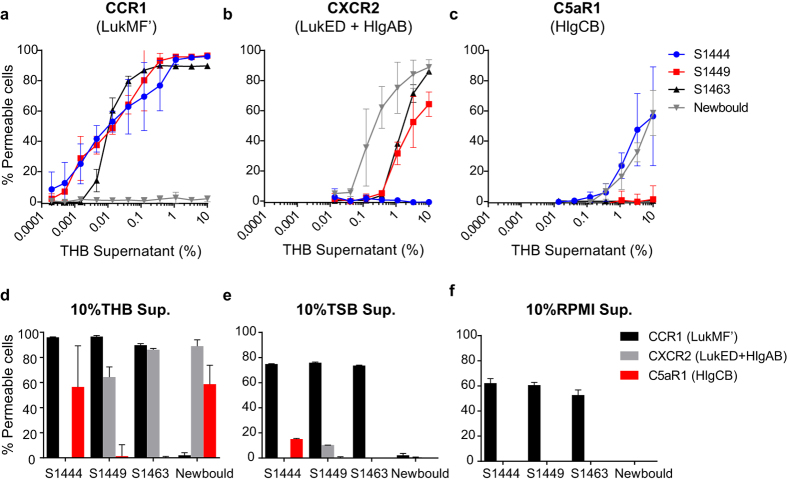
Functional leukocidin production by *S. aureus* mastitis isolates S1444, S1449, S1463, and Newbould. HEK293T cells expressing bovine CCR1 (**a**), CXCR2 (**b**), and C5aR1 (**c**) were incubated with serial dilutions of overnight culture supernatant of S1444, S1449, S1463, and Newbould grown in THB and cell permeability was assessed. Mean results ± SD are shown (n = 3). (**d)** Summary of (**a,b**) and (**c**) for 10% THB culture supernatant. Overnight culture supernatants (10%) of S1444, S1449, S1463, and Newbould grown in TSB (**e**) or RPMI (**f** ) were incubated with HEK293T cells expressing bovine CCR1, CXCR2, and C5aR1 and cell permeability was assessed. Mean results ± SD are shown (n = 3). Permeability of CCR1, CXCR2, or C5aR1 expressing HEK293T cells indicates functional expression of LukMF′, HlgAB and LukED, or HlgCB, respectively.

Because LukMF′, LukED, HlgAB, and HlgCB specifically permeabilise cells carrying their designated target receptors, we were able to use the CCR1, CXCR2, and C5aR1 expressing cells to quantify the secretion of functional leukocidins into the culture supernatant of bovine mastitis isolates^10^. We used CXCR2 and C5aR1 for this assay, because they are the only receptors for the γ-hemolysins and LukED that are not targeted by LukMF^21^. Presence of the *hlgAB, hlgCB, lukED* operons was confirmed in all 10 strains by PCR, while LukMF′ was present in 6 of the strains (Table 1). S1444, S1449, and S1463 harbouring the *lukMF*′ operon secrete high levels of functional LukMF′ in Todd Hewitt Broth (THB), as indicated by the efficient permeabilisation of CCR1 expressing cells (Fig. 2a). As expected, supernatant of Newbould, a strain lacking the *lukMF*′ operon, was unable to permeabilise the CCR1 expressing cells. However, Newbould supernatant kills CXCR2 and C5aR1 expressing cells through secretion of functional HlgAB/LukED and HlgCB (Fig. 2b,c). Strikingly, S1444 does not secrete detectable levels of functional HlgAB or LukED in our assay, while S1449 and S1463 lack measurable levels of HlgCB. Next, we analysed the leukocidin secretion profile of S1444, S1449, S1463 and Newbould grown in in Tryptic Soy Broth (TSB) (Fig. 2e) and RPMI (Fig. 2f) as compared to THB (Fig. 2d). Remarkably, while levels of the other leukocidins were low or undetectable even at relatively high (10%) supernatant percentages, LukMF′ was highly secreted in TSB and RPMI as well. This is in line with previously published results of the promoter activity for the S1444 strain grown in different media^21^.Therefore, we used THB supernatants for all further experiments. In total, THB culture supernatants of 10 mastitis isolates were analysed and results are displayed in Table 1. Values obtained with the C5aR1 expressing cells were corrected for the reduced susceptibility of this cell line for purified toxin. Functional LukMF′ secretion was observed for all strains carrying this operon, while HlgAB/CB and LukED expression was more variable among strains. Overall, LukMF′ secretion induced the highest level of pore formation in HEK293T cells expressing its specific receptor, indicating that LukMF′, when encoded by mastitis isolates, is the most abundantly secreted leukocidin *in vitro* under diverse growth conditions.

### LukMF′ expression levels correlate with neutrophil toxicity

Since expression levels of LukMF′ are the highest of all leukocidins secreted by bovine mastitis isolates, we set out to further specify the importance of this toxin as a cytotoxic agent for bovine neutrophils. Quantification of LukM secretion in overnight THB culture supernatants by ELISA demonstrated variable secretion of LukM by LukMF′ positive strains (Table 1). Culture supernatants of LukMF′ positive strains were more toxic towards bovine neutrophils than LukMF′ negative strains and toxicity appeared to correlate with LukM secretion (Table 1).

**Figure 3:**
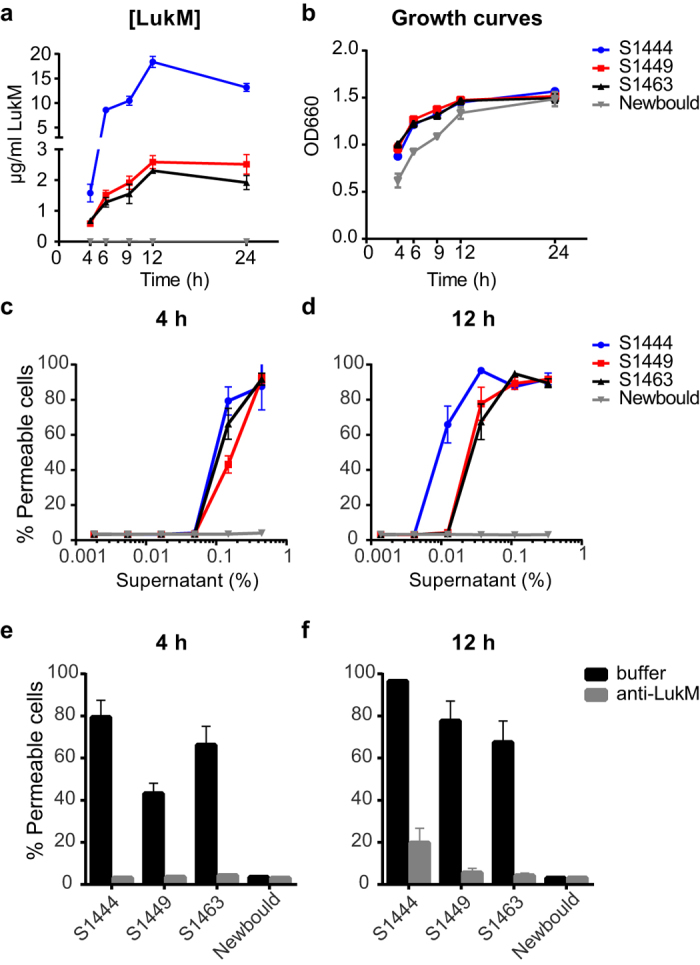
Levels of *in vitro* secreted LukMF′ correlate with neutrophil toxicity of supernatant. (**a**) Protein levels of LukM in culture supernatant of S1444, S1449, S1463, and Newbould grown in THB as measured by capture ELISA at different time points. Mean results ± SD from three independent cultures are shown. (**b**) Growth curves of the cultures from (**a**) measured as the optical density at 660 nm (OD660). Bovine neutrophils were treated with the same supernatant of S1444, S1449, S1463, and Newbould obtained at 4 h (**c**) or 12 h (**d**) of culture in THB and cell permeability was assessed. Bovine neutrophils were exposed to 0.15% 4 h culture supernatant (**e**) or 0.04% 12 h culture supernatant (**f** ) of S1444, S1449, S1463, and Newbould pre-incubated with buffer or 10 μg/ml anti-LukM monoclonal antibody (anti-LukM). Pore formation was measured and mean results ± SD are shown for the three independent culture supernatants. Data for one donor is depicted and is representative for three different animals.

We selected three LukMF′-positive strains with a different toxin secretion profile in THB (S1444, S1449, and S1463) to study LukM expression and its effect on neutrophils in more detail and compared the results to Newbould as a LukMF′-negative control strain. First, we analysed the kinetics and levels of LukM production in THB culture supernatant of these strains at different time points. LukM could readily be detected after 4 h of growth at concentrations of 0.6 μg/ml for S1449, 0.7 μg/ml S1463 and 1.6 μg/ml for S1444 (Fig. 3a,b). LukM levels in supernatant showed a sharp increase between 4 and 12 h of culture, especially for strain S1444, after which LukM levels reached a plateau for all three strains. This resulted in major differences in LukM levels between the strains at 12 h, with S1449 and S1463 producing 2.6 and 2.3 μg/ml respectively, whereas S1444 reached levels of 18.4 μg/ml. As expected, LukM was not detected in culture supernatant of Newbould.

Next, we tested if the observed differences in LukM levels influenced the cytotoxicity of the supernatants towards bovine neutrophils. The 4 h culture supernatant of S1444 was already more potent at killing bovine neutrophils as compared to S1449 and S1463 (Fig. 3c) (p ≤ 0.05, Supplementary Fig. S1). In accordance with the steep incline of LukM levels in S1444 in this culture period, these differences became even more profound at 12 h (Fig. 3d) (p ≤ 0.01, Supplementary Fig. S1). For S1444, it was already reported that LukMF′ is the major secreted cytotoxic agent *in vitro* and *ex vivo*^21^. However, for other LukMF′-positive mastitis isolates, the relative contribution of LukMF′ to neutrophil cytotoxicity is unknown. In order to determine this for S1449 and S1463, we incubated all supernatants with a neutralizing anti-LukM monoclonal antibody before incubating them with neutrophils. The monoclonal antibody effectively blocked the permeabilisation of neutrophils by the supernatant of S1444, whereas an isotype control antibody had no effect (Supplementary Fig. S2). Pre-treatment of the supernatants of all four strains with anti-LukM, confirmed that LukMF′ is indeed the major secreted toxin affecting bovine neutrophils in culture supernatant of the mastitis isolates S1444, S1449, and S1463 (Fig. 3e,f).

In summary, we showed that LukMF′ is differentially produced by *S. aureus* mastitis isolates *in vitro* and that LukM levels in culture supernatant correlate with neutrophil cytotoxicity. Finally, by applying a neutralizing monoclonal antibody, we confirmed that LukMF′ is the major neutrophil killing agent secreted by bovine mastitis isolates.

### LukM is produced *in vivo* during experimental infection and is associated with severity of mastitis

**Figure 4:**
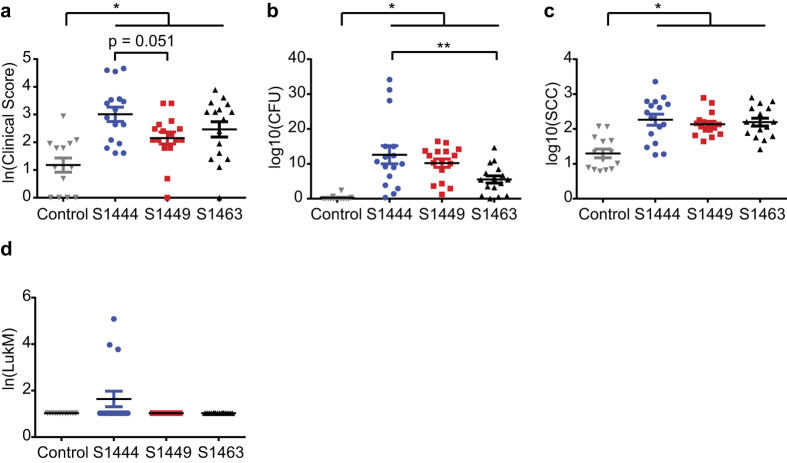
Intramammary challenge with LukMF′ positive *S. aureus* strains. Cumulative measurements of signs of an *S. aureus* infection at quarter level during the 22 day challenge period. Mean ± SEM are shown. (**a**) Clinical score, based on scoring clinical signs of mastitis on the quarter and on milk. (**b**) *S. aureus* Colony Forming Units (CFU) in milk. (**c**) Somatic cell count (SCC) in milk. (**d**) LukM concentration in milk in ng/ml. Data was analysed using linear mixed effect models with cow as a random factor: *P < 0.05, **P < 0.01.

To study the role of LukMF′ *in vivo*, cows were challenged intramammarily in the left and right front quarters of the udder with high (S1444) or intermediate (S1449, S1463) LukMF′ producing *S. aureus* isolates (Supplementary Table S2). Building on the *in vitro* results, we hypothesized that mastitis would be most severe in quarters challenged with the high LukMF′ producing *S. aureus* isolate. Figure 4 shows the clinical score (detailed in the Methods section), CFU, milk somatic cell count (SCC), and LukM concentration at quarter level summed (or averaged at quarter level for SCC) across the challenge period. The clinical scores were most severe within the group challenged with the high LukMF′ producing S1444 strain (Fig. 4a). The linear mixed effect (LME) statistical model with challenge group alone as predicting variable showed the best fit with the clinical score (Supplementary Table S3). The estimate for the S1444 challenged quarters was the highest, i.e. after taking into account the fixed and the random effects the model estimates the clinical score to be highest in the S1444 group, and there was a tendency (p = 0.051) for a higher estimate compared to S1449 (Model A, Table 2). The highest CFU’s in milk were measured in the S1444 challenged quarters (Fig. 4b). The best model for CFU included challenge group as the independent variable (Supplementary Table S3). For this LME model (Model B, Table 2) the estimate for the S1444 challenged quarters was the highest and was significantly higher than for S1463 (p = 0.008). In dairy cows, inflammation of the udder is monitored by measuring the SCC, which reflects the migration of neutrophils to the udder. The SCC was increased in the challenged quarters (Fig. 4c) and challenge group and SCC at challenge were included in the best model for the average SCC during the challenge (Supplementary Table S3). The SCC was significantly higher in the *S. aureus* challenged quarters than in the control quarters, but was comparable between the *S. aureus* challenge groups (Model C, Table 2).

Flow cytometry of milk cells on day 3 post challenge confirmed that neutrophils were the major cell type migrating into the milk and showed that at day 3 the live/dead ratio of milk cells was similar in all animals (data not shown). Naturally occurring bovine antibodies specific for LukM have been shown to neutralize LukMF′ killing *in vitro*^26^, therefore, antibody levels against LukM at challenge were included in the models. Since IgG1 is much more abundant in milk than IgG2^27^, the effects of IgG1 and IgG2 LukM specific antibody levels were tested separately. However, there was no effect of LukM antibody levels at challenge on clinical score, CFU or SCC (Supplementary Table S3). Comparison of LukM antibody levels before and after challenge indicated that LukM antibody levels tended to increase more in animals with severe mastitis (Supplementary Fig. S3).

**Figure 5:**
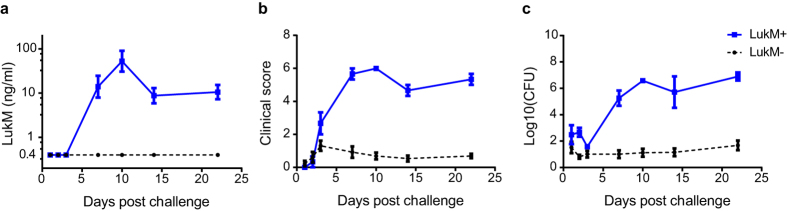
LukM levels in milk correlate with the severity of mastitis in S1444 challenged quarters. (**a**) LukM levels in milk, (**b**) clinical score and (**c**) *S. aureus* Colony Forming Units (CFU) in milk grouped according to quarters with detectable (LukM+; n = 3) or undetectable (LukM−; n = 13) levels of LukM in milk. Mean ± SEM are shown.

LukM was detected in the milk of three quarters challenged with the high LukMF′ producing *S. aureus* strain S1444 (Fig. 4d). LukM was first detectable in milk on day 7 and levels remained high in these three quarters throughout the follow up period (Fig. 5a). Within the S1444 challenged quarters the clinical score and CFU were notably higher for the LukM positive quarters compared to the negative quarters (Fig. 5b,c). The LME model including LukM concentration in milk as the only independent variable had a lower AIC value and therefore a better fit with the clinical score than the model with CFU’s in milk (Supplementary Table S4, Table 3), implying that the clinical scores correlated better with LukM concentration in milk than with bacterial load.

Altogether, the results of the experimental infection indicated that the clinical signs of mastitis were most severe and that the *S. aureus* bacterial load was highest in quarters challenged with the high LukMF′ producing strain (S1444). In addition, *in vivo* LukM levels measured in milk during infection correlated with the clinical severity of mastitis.

## Discussion

The phage encoded *S. aureus* leukocidin LukMF′ has previously been shown to be a potent, bovine-specific virulence factor^16^,^21^. However, knowledge on the role of LukMF′ in the pathogenesis of bovine mastitis is limited. In the present study, we showed that bovine *S. aureus* actively secretes LukMF′ *in vivo* during the course of intramammary infection and that LukM levels in milk are associated with the clinical severity of mastitis. Furthermore, we showed that while *in vitro* LukM expression levels differ between strains, LukMF′ is the most abundantly secreted leukocidin and the major neutrophil killing agent of bovine mastitis isolates.

In recent years, the effects of the staphylococcal leukocidins on the murine and human immune system have been studied extensively. Here, we set out to elucidate their importance in the context of bovine mastitis, one of the most important diseases in dairy cattle^2^. While LukED and the γ-hemolysins were already described to have pore forming capacity on bovine neutrophils^13^, our understanding of the bovine receptors employed by these leukocidins was incomplete. We showed that LukED and HlgAB efficiently permeabilze cells expressing bovine CXCR2, which is also the human orthologue target of these toxins^10^. HlgCB has previously been shown to target both bovine C5aR1 and C5aR2^25^. While the γ-hemolysins are unable to target murine CXCR2 and C5aR1/C5aR2, it is striking that the receptor orthologues of natural hosts of *S. aureus* (e.g. humans, cattle and rabbits) are compatible. Conserved domains in the extracellular loops of these receptors presumably explain this compatibility^25^. LukAB stands out from the family of staphylococcal leukocidins, having a low sequence homology with other leukocidins and a distinct mode of action^4^. It appears to be poorly secreted^10^, associates with the cell surface of the bacterium^28^ and is proposed to kill cells from within after phagocytosis^22^,^28^,^29^. Whereas LukAB is toxic for human and rabbit neutrophils^30^, it has very limited effect on murine neutrophils^4^,^31^,^32^. Here, we show that bovine neutrophils were not susceptible to LukAB, even though they express CD11b^33^, the described target receptor of LukAB. LukAB is, therefore, unlikely to play an important role in bovine *S. aureus* disease.

Most bovine *S. aureus* isolates harbour the genes for LukAB, LukED, and the γ-hemolysins, while only a proportion carries the φSa1 phage encoding LukMF′^31^. Barrio *et al*.^13^ compared the biological activity of LukMF′, LukED, and γ-hemolysins that were purified from culture supernatant and showed that LukMF′ was the most potent toxin acting on bovine neutrophils. Using recombinant proteins we found roughly similar results, except that HlgAB was more active than LukMF′. However, the activity of recombinant leukocidins produced in *E. coli* may differ from the true biological activity of leukocidins secreted by *S. aureus* and to overcome this problem we used supernatants directly in our other assays. Besides the biological activity, the pathogenic relevance of leukocidins also depends strongly on expression levels. We quantified and compared the expression and secretion of different leukocidins within and between 10 different *S. aureus* mastitis isolates and demonstrated that LukMF′ was the most abundantly secreted leukocidin. Strikingly, *in vitro* expression of HlgAB, LukED, and HlgCB was highly variable among bovine mastitis isolates and some strains did not produce functional levels of these toxins, i.e. levels sufficient to induce killing of HEK293T cells bearing their cognate receptor. This indicates that there might be inter-strain differences in regulation of expression of these toxins and that their contribution to the pathogenesis of *S. aureus* mastitis could be strain dependent. In contrast, all strains carrying the lukMF′ operon secreted functional LukMF′. Growth conditions and environmental stimuli are known to influence leukocidin expression by *S. aureus*^4^,^34^. Regulators of LukMF′ expression have not yet been identified, but promotor analysis of the S1444 strain revealed that activity of the LukMF′ promoter was significantly higher than all other leukocidin promoters under diverse growth conditions^21^. This corresponds with our finding that LukMF′ is actively and highly secreted in THB and TSB, as well as in the eukaryotic culture medium RPMI.

Using a LukMF′ neutralizing monoclonal antibody, we demonstrated that, *in vitro,* LukMF′ is the major neutrophil killing agent secreted by bovine mastitis isolates carrying this operon. Neutrophils are important effector cells in the first line of defence against *S. aureus*^12^ and we hypothesized that killing of neutrophils by LukMF′ hampers the phagocytic immune response leading to more severe infections. Intramammary challenge of cows with high and low LukMF′ producing *S. aureus* isolates showed a higher bacterial load and a tendency for more severe clinical signs of mastitis in quarters challenged with the high LukMF′ producing isolate. LukM was detectable in the milk of quarters with severe clinical signs of mastitis, showing that *S. aureus* actively secretes LukM during infection. *In vitro,* LukM production correlated with neutrophil killing and, since LukF′ is expressed from the same operon as LukM, we assume that LukM measured in milk reflects production of functional LukMF′. In humans, expression of the phage encoded leukocidin PVL is associated with increased severity of infection^35^,^36^,^37^. Here, we showed that *in vivo* LukM production correlated with the clinical severity of mastitis, suggesting that the phage encoded leukocidin LukMF′ may be important in the pathogenesis of *S. aureus* mastitis. LukMF′-negative strains, such as Newbould, also cause mastitis in cattle, showing that LukMF′ is not a prerequisite for *S. aureus* to cause an intramammary infection and that other toxins or virulence factors also play a role in the pathogenesis of mastitis. However, whereas the LukMF′-negative strain Newbould causes mostly mild clinical symptoms^38^, infections with the LukMF′-positive isolates RF122 and Ch122 are characterised by severe forms of mastitis^38^,^39^. In accordance with our results, Rainard *et al*.^39^ showed that following intramammary challenge of six goats with *S. aureus,* LukMF′ was also detectable in the milk of two goats with severe mastitis. *S. aureus* may colonize the teat skin in cattle and strains present on teat skin can be different from those isolated from milk^40^,^41^. Interestingly, one study found a high prevalence of LukMF′ positive strains on teat skin^41^. More detailed epidemiological studies are needed to find out whether LukMF′ is associated with *S. aureus* carriage and whether carriage of LukMF′ positive strains on the skin is associated with more frequent and/or more severe cases of mastitis. *In vitro*, LukM was expressed within 8 hours when *S. aureus* was cultured in milk^21^. In our challenge study, LukM was first detected in milk on day 7 post challenge, corresponding with the onset of clinical mastitis and strong increase in CFU in milk. LukM levels were probably below the detection limit before day 7, considering that dairy cows produce around 30 liters of milk per day, which leads to strong dilution of the toxin. Bovine serum antibodies have been shown to neutralize LukMF′ killing *in vitro*^26^. However, antibody levels in milk are low as compared to serum and the concentration of LukMF′ at foci of infection may be higher than the limited neutralizing capacity of milk antibodies, which could explain why there was no effect of LukM antibody levels at challenge on the course of infection. Milk samples with detectable levels of LukM also had high CFU counts and this raises the question whether more bacteria simply produce more LukM leading to higher concentrations of LukM in milk or that the production of LukMF′ leads to favourable growth conditions and therefore to higher CFU counts. Since our *in vitro* result indicate LukMF′ is an important virulence factor and the LukM concentration in milk showed a better fit with severity of mastitis than CFU counts, this favours the latter explanation. Future research, e.g. intramammary challenge with wild-type and isogenic LukMF′ knockout strains, is needed to identify the effect of strain differences and unequivocally prove the role of LukMF′ in the pathogenesis of bovine *S. aureus* mastitis. Vaccination with LukMF′ to increase antibody levels in milk may neutralize the toxic effects of LukMF′ during *S. aureus* infection. Alternatively, infusion of a neutralizing monoclonal antibody during infection, e.g. KoMa43 used in this study, may also reduce the severity of *S. aureus* mastitis.

We studied the relevance of secreted *S. aureus* leukocidins in the pathogenesis of mastitis in cattle, a natural host of *S. aureus*. The bovine target receptors of HlgAB and LukED were identified and LukAB was shown to be non-toxic for bovine neutrophils. *In vitro* assays showed LukMF′ was the most abundantly secreted leukocidin and the major neutrophil killing agent of bovine mastitis isolates. We demonstrated that *S. aureus* actively secretes LukM during intramammary infections in cattle and that LukM levels in milk are associated with severity of disease. In conclusion, this report underlines the importance of LukMF′ as a virulence factor in bovine mastitis, both *in vitro* and *in vivo*.

## Methods

### Ethics statement

The use of animals was approved by the Ethical Committee for Animal Experiments of the Utrecht University (Permit No. DEC2012.II.10.152) or an independent ethical committee (MSD Animal Health). Animal experiments were performed in accordance with European Community guidelines and national laws on animal experiments. The use of human venous blood in this study was approved by the medical ethics committee of the UMC Utrecht (Permit No. 07-125/C, 2010). Written informed consent was obtained from all donors in accordance with the Declaration of Helsinki and al experiments were performed in accordance with European Community guidelines and national laws on experiments involving humans.

### Leukocyte isolation

Bovine blood was collected from three healthy Holstein-Friesian cows using a sterile blood collection system with EDTA anti-coagulant (BD Vacutainer). Neutrophils were isolated from bovine blood by Percoll (1.09176 g/l) centrifugation^21^. Human heparin blood was collected from healthy volunteers and the isolation of neutrophils was performed by Ficoll/Histopaque centrifugation^42^. Purity was ≥95% and viability ≥95%, for both human and bovine neutrophils.

### Bacterial strains and culture conditions

*S. aureus* strains used in this study are listed in Table 1 and were isolated from bovine mastitis cases throughout Europe^21^. Strains were cultured in triplicate overnight or for 4, 6, 9, 12, and 24 hours in Todd Hewitt Broth (THB), Tryptic Soy Broth (TSB), or RPMI supplemented with 1% Casamino Acids, and supernatants were harvested by centrifugation. Supernatants were sterilized using 0.2 μm filters and subsequently stored at −20 °C. PCRs for checking the presence of leukocidin S-component genes were performed on genomic DNA isolated according to methods described elsewhere^43^. Primers were designed to be highly gene specific and are listed in Supplementary Table S5^25^.

### Cloning, expression, and purification of recombinant leukocidins

Recombinant HlgA, HlgB, HlgC, LukE, LukD, LukM, LukF′, LukA, and LukB proteins were generated in *E. coli* with a non-cleavable N-terminal 6xHIS tag as described previously^10^,^21^,^44^. In short, coding sequences were cloned into either a slightly modified pRSETB vector (Invitrogen), pQE-30 (Qiagen, Courtaboeuf, France) or pIVEX2.4d (Roche) and expression plasmids were transformed in *E. coli* Rosetta Gami (DE3) plysS or BL21 (DE3). Protein expression was induced with 1 mM Isopropyl β-D-1-iogalactopyranoside (IPTG). Proteins were isolated from a HiTrap chelating HP column under native conditions and eluted using an imidazole gradient. Subsequently, proteins were stored in PBS and purity (>95%) was confirmed using SDS-PAGE electrophoresis. LukA and LukB were eluted from the purification column under denaturating conditions. To prevent precipitation of LukB, the protein was first dialysed to a Tris-arginine buffer, pH10, and subsequently pH was slowly increased towards a physiological pH. Finally, both toxins were dialyzed and stored in a Tris-NaCl buffer.

### Cloning of bovine chemokine receptor expressing plasmids

Bovine chemokine receptor expressing plasmids were prepared according to methods described elsewhere^21^. In short, mRNA was isolated from bovine blood using an Ambion RiboPure-Blood kit (Life Technologies) and cDNA was prepared with the SuperScript III First-Strand Synthesis System (Invitrogen). Bovine CXCR1, CXCR2, CXCR3, CXCR4, CXCR6, XCR1, CX_3_CR1, CCR1, CCR2, CCR3, CCR4, CCR5, CCR6, CCR7, CCR8, CCR9, C5aR1, C3aR, PAFR, P2Y14R, CCR1L, CCR2L, and CMKLR1 were amplified from bovine cDNA by PCR using PfuTurbo DNA polymerase (Stratagene) and subsequently cloned into the pcDNA3.1 vector (Invitrogen) for transient expression as described previously^21^,^24^. In addition, bovine CXCR2, CCR1, and C5aR1 cDNA were cloned into the pIRESpuro3 vector (Clontech) for stable expression^10^.

### Cell lines and Transfections

HEK293T cells (a human embryonic kidney cell line) were obtained from the American Type Culture Collection. Cells were maintained in Dulbecco’s modified Eagle’s medium (DMEM) supplemented with 10% FCS and 100 U/ml penicillin and 100 μg/ml streptomycin. Transfections were performed according to the manufacturer’s protocol using 4 μg DNA and 5 μl Lipofectamine 2000 (Life Technologies). For transient transfections with pcDNA3.1 plasmids containing bovine chemokine receptor cDNA, cells were harvested after 24 hours using 0.05% Trypsin/0.53 mM EDTA and subsequently used in cell permeabilisation assays. For stable transfections of bovine CCR1, CXCR2, and C5aR1 using the pIRESpuro3 vector, HEK293T cells were put under selection pressure after 24 hours using 1 mg/ml puromycin in the DMEM medium described above^10^. Surviving cells were subsequently subcloned after several weeks of culture to generate HEK293T cells stably expressing CCR1, CXCR2 or C5aR1.

### Cell permeabilisation assays

Cell lines and neutrophils (3 × 10^6^ cells/ml) were incubated for 30 minutes with recombinant toxin (HlgAB, HlgCB, LukED, LukAB, or LukMF′) at 37 °C, 5% CO_2_ in a volume of 50 μl in RPMI containing 0.05% human serum albumin (Sanquin). Incubation with bacterial supernatants were performed accordingly, with the exception that cell lines were incubated with supernatant for 15 minutes and neutrophils for 30 minutes, both in the presence of 10% fetal bovine serum to neutralize phenol-soluble modulins^45^. To block LukMF′ activity, supernatants were pre-incubated for 30 min at room temperature with mouse anti-LukM monoclonal antibody (KoMa43, Podiceps, The Netherlands^21^) or isotype control at a final concentration of 10 μg/ml, before being added to the cells. Cells were analysed by flow cytometry and pore formation was defined as intracellular staining by 4′,6-diamidino-2-phenylindole (DAPI). S- and F-components were used in equimolar concentrations in all assays described. For analysis, the percentage of spontaneously permeabilised HEK293T cells was subtracted from the percentage of permeable cells following incubation with toxin. To compare the functional leukocidin production between strains, the lowest concentration of culture supernatant at which > 50% of the HEK293T cells expressing either CCR1, C5aR1, or CXCR2 became permeable to DAPI after 15 minutes were scored on a categorical scale based on supernatant percentage thresholds. In order to compare the results obtained with the different cell lines and leukocidins, results for the HEK293T-C5aR1 cell line incubated with HlgCB were corrected by dividing the supernatant percentage thresholds by 3.9, since the EC50 for this cell line-toxin combination was 3.9 fold higher than the average EC50 of the other combinations.

### LukM antigen and LukM specific antibody ELISA

To detect LukM, NUNC MaxiSorp ELISA plates were coated overnight with 32 μg/ml polyclonal Bovine IgG. Bovine IgG was isolated from the colostrum of a cow with high antibody levels against LukM by liquid affinity chromatography using Protein G columns, in 0.1 M sodium-bicarbonate buffer (pH9.6). Blocking was performed with 4% skimmed milk in PBS/0.05%Tween20. Subsequently, *S. aureus* culture supernatants, milk or recombinant LukM were added. Next, 3 μg/ml of Mouse anti-LukM monoclonal antibody (KoMa43, Podiceps, The Netherlands) was added to the wells and bound anti-LukM monoclonal antibody was detected with HRP-conjugated Goat anti-Mouse IgG (BioLegend). Wells were incubated with tetramethylbenzidine (TMB) as a substrate, reactions were stopped by adding 4 N sulphuric acid and extinctions (450 nm) were measured on a Microplate Reader. Milk samples were pretreated at 95 °C for 10 minutes to prevent interference from antibodies in the milk. Antibodies, recombinant proteins and samples were diluted in 1% skimmed milk in PBS/0.05%Tween20. All incubations were performed on a shaker at room temperature, except for coating which was performed overnight at 4 °C. In between incubation steps, wells were washed with PBS/0.05%Tween20. All samples were measured in duplicate and LukM concentrations were calculated from recombinant LukM standard curves. The detection limit of LukM in milk was 0.4 ng/ml.

To detect LukM specific antibodies, ELISA plates were coated with 1.25 μg/ml recombinant LukM. Blocking was performed with blocking reagent for ELISA (Roche). Serum was diluted 1/2000 and 1/20.000 for detection of IgG1 and IgG2, respectively. Next, bound bovine IgG1 and IgG2 was detected with the monoclonal antibodies Mouse anti-Bovine IgG1 15.8.1a (Prionics) and Mouse anti-Bovine IgG2 12.5.4c (Prionics), respectively. Antibodies and samples were diluted in blocking reagent for ELISA. Other ELISA conditions were the same as described above. Positive control samples were measured in quadruplicate and sample to positive ratios (S/P) were calculated from duplicate measurements.

### Intramammary challenge of cattle with *S. aureus*

In total 31 clinically healthy mid-lactation Holstein-Frisian heifers purchased in Germany were allotted to 4 groups (Supplementary Table S2). Animals were challenged in the left and right front quarter of the udder and, since quarters are anatomically and functionally separate units, a quarter was regarded as the experimental unit in this study. During the 7 day acclimatization period, bacterial culture of quarter milk was performed to confirm that none of the animals had an *S. aureus* infection pre-challenge. Animals assigned to the control group were not infected. Inocula of S1444, S1449, and S1463 were prepared by diluting bacterial glycerol stock into a 0.9% NaCl solution. Before challenge teat ends were disinfected with 70% alcohol. Two to three hours after the morning milking the left and right front quarters were infused with 1 ml inoculum, containing approximately 500 CFU, using sterile plastic infusion cannulas attached to a syringe. The exact inoculum dose was checked by culturing on blood agar plates and was within a 1.5 fold margin of the intended CFU dose of 500. Clinical signs of mastitis were scored daily at quarter level by scoring the appearance of the quarter and the milk. Quarters were scored according to: 0 = soft, pliable udder, no abnormalities; 1 = slight swelling; 2 = moderate swelling, warm skin; 3 = severe swelling and warm skin. Milk was scored according to: 0 = normal milk; 1 = milk with some flakes or clots (<10); 2 = milk with many flakes or clots (≥10); 3 = mastitic milk (serous, watery, severe cloths or purulent). Addition of the quarter and milk scores resulted in the clinical score. Milk collected during the morning milking at days 0, 1, 2, 3, 7, 10, 14, 22 was used for bacteriological examination, somatic cell count measurements and the LukM antigen ELISA. For bacterial examination 50 μl of milk or tenfold dilutions were incubated on blood agar plates for 24 hours at 37 °C and *S. aureus* colony counts were used to calculate the CFU per 50 μl milk. Milk somatic cell counts (SCC) were determined by a commercial milk quality assurance laboratory (Qlip, Zutphen, The Netherlands). All CFU counts and SCC were base-ten log transformed. At day three post challenge, cells in the milk were analysed by flow cytometry. Neutrophils and mononuclear cells were discriminated based on forward and sideward scatter^46^,^47^ and live and dead cells were discriminated with DAPI staining. Serum samples were collected just before challenge at day 0 and at 22 days post challenge.

### Statistical analysis

Flow cytometric data was analysed with Flow Jo (Tree Star Software). Half maximal lytic concentrations (EC50) were calculated using nonlinear regression analyses and differences in EC50 between groups were compared by a One-Way ANOVA followed by Tukey’s post-hoc test (Prism 6, GraphPad Software).

The intrammary *S. aureus* challenge was analysed with linear mixed models in SPSS 22 (IBM). To account for the repeated measures within cows at quarter level, cow was included as a random effect with variance components as covariance structure. For the analysis of the whole dataset the clinical score and the CFU at quarter level were summed and the SCCs at quarter level were averaged over the 22 day challenge period resulting in the dependent variables. The summed clinical score was normalized using a natural log transformation. Challenge group, SCC at challenge, and LukM IgG1 and IgG2 antibody levels at challenge were included as independent variables. To analyse the data within the S1444 challenged animals, the dependent variable clinical score and the independent variables LukM concentration in milk and CFU were not summed but included as repeated measures for days 1, 2, 3, 7, 10, 14, and 22 with a first-order autoregressive covariance structure. Cow was again included as a random effect and the LukM concentration in milk was transformed with a natural log. Models were compared using Akaike information criterion (AIC). First the effect of each individual independent variable on the model was tested. Next, all variables that resulted in a model with an AIC lower than the intercept only model were used for full factorial model building. The model with the lowest AIC that was also 2 points lower than a model with one less variable was selected as the best model for that independent variable. The F-test was used to test whether individual variables improved the fit of the model and Fishers Least Significant Difference test was used to compare the estimates within a variable or to compare the estimate of the variable to the reference category. Plots were constructed to analyse the normality and homoscedasticity of the residuals of the final models.

### Data availability

The authors declare that the data supporting the findings of this study are available within the paper and its supplementary information files, except for the flow cytometry results of milk cells on day 3 post challenge. All data are available from the corresponding author upon reasonable request.

## Additional Information

**How to cite this article**: Vrieling, M. *et al*. LukMF′ is the major secreted leukocidin of bovine *Staphylococcus aureus* and is produced *in vivo* during bovine mastitis. *Sci. Rep.* **6**, 37759; doi: 10.1038/srep37759 (2016).

**Publisher's note:** Springer Nature remains neutral with regard to jurisdictional claims in published maps and institutional affiliations.

## Supplementary Material

Supplementary Information

## Figures and Tables

**Table 1:** Functional leukocidin production by bovine mastitis isolates grown in THB.

Isolate	Country of isolation	LukMF′ PCR^a^	[LukM]^b^	HEK CCR1^c,^^d^	HEK CXCR2^c,^^e^	HEK C5aR1^c,^^f^,*	Bovine neutrophils^g^
S1444	Germany	+	8.2	++++	−	+	+++
S1449	France	+	1.9	+++	+	−	++
S1452	Netherlands	+	27.7	++++	+++	++	+++
S1463	France	+	1.8	++++	+	−	++
RF122	Ireland	+	1.5	+++	++	−	++
P83	Germany	+	1.3	+++	++	+	+++
Newbould	Canada	−	0.0	−	++	+	−
S1448	Spain	−	0.0	−	++	+++	++
S1456	Germany	−	0.0	−	−	−	−
S1461	Netherlands	−	0.0	−	+++	++	+

^a^Presence of the *lukMF*′ operon detected by PCR. The *hlgAB, hlgCB, lukED* operons were detected in all isolates.

^b^LukM concentration in overnight culture supernatant in μg/ml determined by ELISA. ^c^Lowest concentration of culture supernatant at which >50% of the cells became permeable to DAPI after 15 minutes incubation. ++++ = 0.01%; +++ = 0.1%; ++ = 1%; + = 10%; − = <50% of the counted cells are DAPI positive at 10% supernatant.

^d^HEK293T cells stably expressing a specific receptor of LukMF′,

^e^HlgAB and LukED or

^f^HlgCB.

^g^Lowest concentration of culture supernatant at which >50% of bovine neutrophils became permeable to DAPI after 30 minutes incubation. +++ = 0.01%; ++ = 0.03%; + = 0.1%; − = <50% of the counted cells are DAPI positive at 0.1% supernatant.

^*^Relative permeability of cells. Supernatant percentage thresholds. Were corrected for the lower EC50 value of recombinant HlgCB.

**Table 2:** Summarizing results of the best models for the dependent variables Clinical Score, Colony Forming Units (CFU) and Somatic cell count (SCC).

Model	Variable^a^	F-test^b^	Estimate	95% CI^c^		P-value^d^		
A: Clinical score ^~^ Group	Intercept	0.000	1.18	0.53	1.83	0.001		
	Group	0.002						
	Control		Reference			Control	S1444	S1449
	S1444		1.83	0.94	2.72	0.000		
	S1449		0.98	0.09	1.86	0.032	0.051	
	S1463		1.29	0.40	2.17	0.006	0.203	0.468
B: CFU ^~^ Group	Intercept	0.000	0.23	3.60	4.07	0.902		
	Group	0.000						
	Control		Reference			Control	S1444	S1449
	S1444		12.36	7.11	17.61	0.000		
	S1449		9.99	4.74	15.24	0.001	0.348	
	S1463		5.28	0.03	10.53	0.049	0.008	0.068
C: SCC ^~^ Group + SCC_challenge_	Intercept	0.000	0.75	0.35	1.15	0.000		
	SCC_challenge_	0.000	0.49	0.24	0.74	0.000		
	Group	0.000						
	Control		Reference			Control	S1444	S1449
	S1444		1.01	0.62	1.41	0.000		
	S1449		0.99	0.59	1.40	0.000	0.924	
	S1463		0.88	0.49	1.28	0.000	0.497	0.568

^a^Group - challenge group with categories Control, S1444, S1449, S1463. SCCchallenge - Somatic cell count at challenge.

^b^F-test of the variable.

^c^95% confidence interval of the estimate.

^d^Fishers Least Significant Difference test of the estimate.

**Table 3:** Summarizing results for the model Clinical Score **~** LukM within the S1444 challenge data.

Variable^a^	F test^b^	Estimate	95% CI^c^		P-value^d^
Intercept	0.000	1.77	1.29	2.25	0.000
LukM	0.000	1.00	0.79	1.22	0.000

^a^LukM – Natural log of [LukM] in milk in ng/ml.

^b,c,d^As in Table 2.
