# Supplementary material for: LukMF′ is the major secreted leukocidin of bovine Staphylococcus aureus and is produced in vivo during bovine mastitis
Source: Sci Rep. 2016 Nov 25;6:37759. doi: 10.1038/srep37759 (PMC5123576; doi:10.1038/srep37759)
Supplement: Supplementary Information [file srep37759-s1.pdf]

LukMF' is the major secreted leukocidin of bovine *Staphylococcus aureus* and is produced *in vivo* during bovine mastitis

Manouk Vrieling[a,b,*], *Eveline M. Boerhout*[c], *Glenn F. van Wigcheren*[a], *Kirsten J. Koymans*[a], *Tanja G. Mols-Vorstermans*[c], *Carla J.C. de Haas*[a], *Piet C. Aerts*[a], *Ineke J.J.M. Daemen*[d], *Kok P.M. van Kessel*[a], *Ad P. Koets*[d,e], *Victor P.M.G. Rutten*[b,f], *Piet J.M. Nuijten*[c], *Jos A.G. van Strijp*[a], *Lindert Benedictus*[a,b]

[a]Department of Medical Microbiology, University Medical Center Utrecht, PO G04.614, Heidelberglaan 100, 3584 CX, Utrecht, The Netherlands

[b]Department of Infectious Diseases and Immunology, Faculty of Veterinary Medicine, Utrecht University, Yalelaan 1, 3584 CL Utrecht, The Netherlands

[c]Ruminant Research and Development, MSD Animal Health, Wim de Körverstraat 35, 5830 AA Boxmeer, The Netherlands

[d]Department of Farm Animal Health, Faculty of Veterinary Medicine, Utrecht University, Yalelaan 7, 3584 CL Utrecht, The Netherlands

[e]Department of Bacteriology and Epidemiology, Central Veterinary Institute part of Wageningen UR, Edelhertweg 15, 8219 PH Lelystad, The Netherlands

[f]Department of Veterinary Tropical Diseases, Faculty of Veterinary Science, University of Pretoria, Private Bag X04, Onderstepoort 0110, South Africa

[*]Corresponding author: m.vrieling@umcutrecht.nl

Supplementary Figures

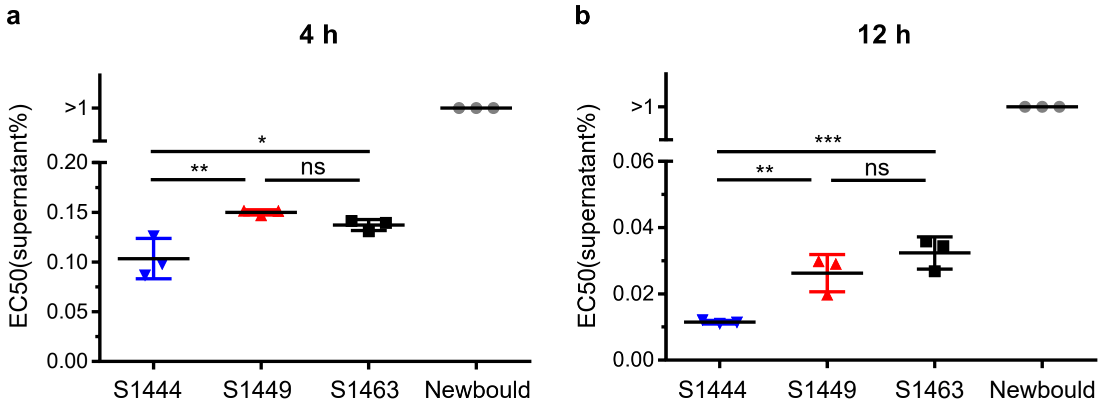

Supplementary Figure S1: Levels of in vitro secreted LukMF' correlate with neutrophil toxicity of supernatant. Bovine neutrophils were treated with supernatant of S1444, S1449, S1463, and Newbould obtained at 4 h (a) or 12 h (b) of culture in THB and cell permeability was assessed. Half maximal lytic concentrations (50% effective concentrations [EC50s]) were calculated for each strain and compared using a One-Way ANOVA followed by Tukey's post-hoc test. Mean results ± SD from three independent cultures are shown. NS not significant, * P≤0.05, *** P≤0.001, **** P≤<0.0001.

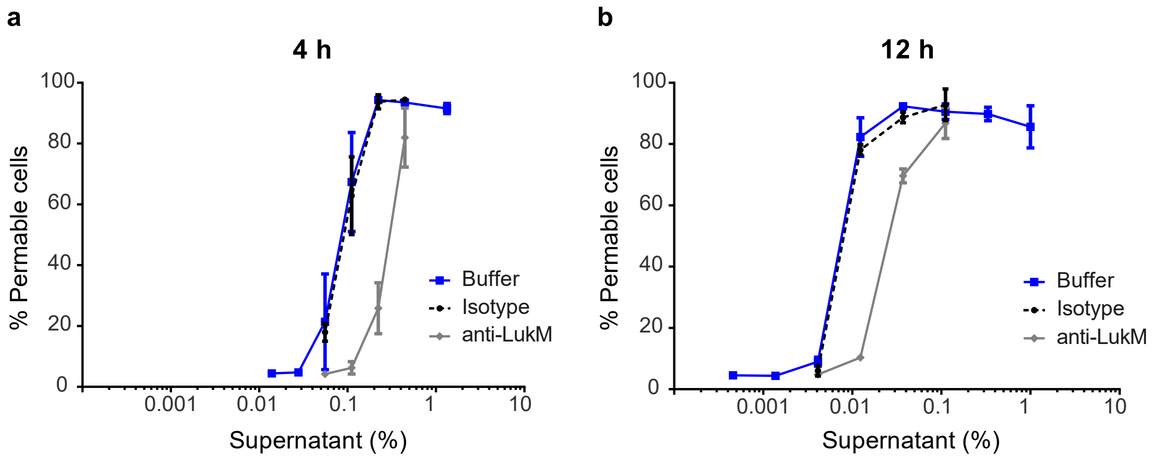

Supplemental Figure S2: Neutrophil cytotoxicity of S1444 supernatant is blocked by a LukM specific monoclonal antibody. Bovine neutrophils were exposed to 4 h (**a**) and 12 h (**b**) culture supernatant of S1444 that were pre-incubated with 10 µg/ml anti-LukM monoclonal antibody (anti-LukM), an isotype control or buffer. Pore formation was measured and mean results ± SD for three independent culture supernatants are shown.

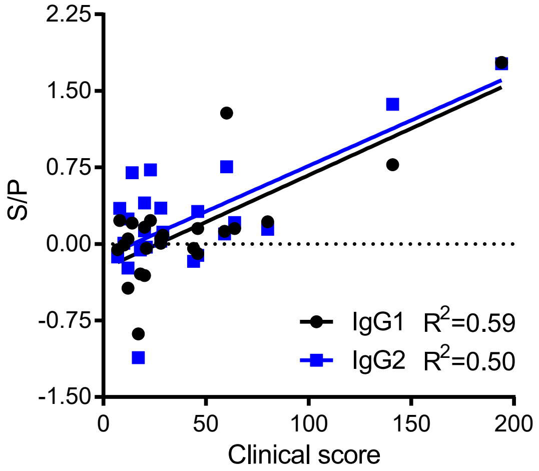

Supplemental Figure S3: Changes in LukM serum antibody levels after intramammary challenge with LukMF' positive *S. aureus* strains. Correlation between the summed clinical score per animal and the difference in IgG1 and IgG2 LukM serum antibody levels between the start (day 0) and the end of the challenge (day 22). Severe mastitis cases tended to have a bigger rise in LukM antibody levels.

S/P sample to positive control ratio.

Supplementary Tables

Supplementary Table S1. Quantitative assessment of pore formation in transiently transfected HEK293T cells upon incubation with different leukocidins. A three times increase of the % of permeable cells as compared to background (no toxin) was considered as the threshold for the induction of pore formation. Transfected cells were incubated with 20 nM and 200 nM of leukocidin and receptors were identified as major receptors (+) when pore formation was induced at 20 nM and as putative minor receptors (-/+) when pore formation was detected at 200nM only.

Receptor	Acc. Nr.	HlgAB	HlgCB	LukED
Bovine CXCR1	NM_001105038.1	-/+	-	
Bovine CXCR2	NM_001101285.1	+	-	+
Bovine CXCR3	NM_001011673.1	-	-	
Bovine CXCR4	NM_174301.3	+	-	
Bovine CXCR6	NM_001014859.1	-	-	
Bovine XCR1	NM_001194965.1	-	-	
Bovine CX_3CR1	NM_001102558.2	-	-	
Bovine CCR1	NM_001077839.1	-	-/+	-
Bovine CCR1L	NM_001075921.1	-	-	
Bovine CCR2	NM_001194959.1	+	-	
Bovine CCRL2L	NM_001075732.2	-	-	
Bovine CCR3	NM_001194960.1	-	-	
Bovine CCR4	NM_001100293.1	-	-	
Bovine CCR5	NM_001011672.2	-	-	+
Bovine CCR6	NM_001194961.1	-	-	
Bovine CCR7	NM_001024930.3	-	-	
Bovine CCR8	NM_001194962.1	-	-	
Bovine CCR9	NM_001098068.1	-	-	
Bovine C5aR1	NM_001007810.3	-	+	-
Bovine C5aR2	NM_001077947.1		+	
Bovine C3aR	NM_001083752.1	-	-/+	
Bovine PAFR	NM_001040538.1	-	-	
Bovine P2Y14R	NM_001077009.1	-	-	
Bovine CMKLR1	NM_001145235.1	-	-/+	

Supplementary Table S2. Intramammary challenge with LukMF' positive *S. aureus* strains.

Group	Challenge strain	Animals / Quarters	Intrammary challenge	[LukM] production *in vitro* (ug/ml)[a]
1	S1444	8 / 16	Front quarters	**18.4**
2	S1449	8 / 16	Front quarters	**2.6**
3	S1463	8 / 16	Front quarters	**2.3**
4	Control	7 / 14	Control, not infected	-

[a] In supernatant after 12 hour culture

Supplementary Table S3. Comparison of the linear mixed models for the *S. aureus* challenge data using Akaike Information Criteria. Models with different (combinations of) fixed effects were tested and for each dependent variable the model with the best AIC is emphasized in bold.

	Dependent variable[b]		
**Fixed effects[a] – Single**	Clinical score	CFU	SCC
Intercept	181.353	417.437	98.912
Group	**172.819**	**404.014**	85.110
$SCC_{challenge}$	183.025	417.006	91.726
LukM IgG1	180.279	418.756	99.143
LukM IgG2	182.78	419.22	100.735
**Fixed effects - Factorial**			
Group + LukM IgG1	173.332		
Group + LukM IgG1 + Group*LukM IgG1	178.590		
Group + $SCC_{challenge}$		404.295	**73.762**
Group + $SCC_{challenge}$ + Group* $SCC_{challenge}$		407.196	73.202

[a] Group - Challenge group, $SCC_{challenge}$ - Somatic cell count at challenge, LukM IgG1/IgG2 - IgG1/IgG2 serum antibody levels at challenge.

[b] Cumulative measurements during the 22 day challenge period of the scoring of clinical signs of mastitis on the quarter and on milk (clinical score), *S. aureus* Colony Forming Units in milk (CFU) and the average Somatic cell count in milk (SCC) at quarter level.

Supplementary Table S4. Comparison of the linear mixed models for the dependent variable Clinical Score within the S1444 challenge data using Akaike Information Criteria (AIC).

Fixed effects[a]	AIC
Intercept	360.462
LukM	**314.779**[b]
CFU	346.482
LukM + CFU	315.701
LukM + CFU + LukM*CFU	317.536

[a] LukM – Natural log of [LukM] in milk in ng/ml. CFU - *S. aureus* Colony Forming Units in milk
[b] Model with the lowest AIC.

Supplementary Table S5. Primers used to identify leukocidin S-component genes in the genome of bovine *S. aureus* mastitis isolates.

Gene	Primers
hlgA	Fwd: 5'---CACAAGACCCAACTGGTCCAGCAGC---3'
	Rev: 5'---GCTAAACGATGTCTTGTCACGTAAGC---3'
hlgC	Fwd: 5'---GGAAGCGATATAGAAATTATC---3'
	Rev: 5'---TAAATCGCTATCAAAGGCTG---3'
lukE	Fwd: 5'---AACGAATGATTTGGCCATTC---3'
	Rev: 5'---CCAGTTCTAGGGAATAAAGTCGCATATG---3'
lukA	Fwd: 5'---GTTATCAGCAGCAACGACTC---3'
	Rev: 5'---CAATTCTCGTATTTCTATAG---3'
lukM	Fwd: 5'---CGGGATCCACTACTAATGCAGAAGATATTGGCGACGA---3'
	Rev: 5'---ATATGCGGCCGCTTAGTTGTGCCCCTTTACTTTAATTTCGTAC---3'
